# Asynchrony among insect pollinator groups and flowering plants with elevation

**DOI:** 10.1038/s41598-020-70055-5

**Published:** 2020-08-06

**Authors:** Opeyemi Adedoja, Temitope Kehinde, Michael J. Samways

**Affiliations:** 1 Department of Conservation Ecology and Entomology, Stellenbosch University, Stellenbosch, South Africa; 2 Department of Conservation and Marine Sciences, Cape Peninsula University of Technology, Cape Town, South Africa; 3 Department of Zoology, Obafemi Awolowo University, Ile-Ife, Nigeria

**Keywords:** Biogeography, Ecosystem ecology

## Abstract

Mountains influence species distribution through differing climate variables associated with increasing elevation. These factors determine species niche ranges and phenology. Although the distribution patterns of some specific insect groups relative to elevation have been determined, how differing environmental conditions across elevation zones differentially influence the phenology of various insect groups is largely unknown. This is important in this era of rapid climate change. We assess here how species composition and seasonal peaks in abundance among different insect pollinator groups and flowering plants differ across four floristically distinct elevation zones up a sentinel mountain subject to strong weather events. We sampled insect pollinators in four major groups (bees, wasps, beetles and flies) over two spring seasons. Pollinator species composition across all elevation zones tracks flowering plant species composition. In terms of abundance, beetles were the dominant group across the three lower zones, but declined greatly in the summit zone, where flies and bees were more abundant. Bee abundance peaked earlier than the other groups across all four elevation zones, where there were significant peaks in abundance. Bee abundance peaked earlier than flowering plants at the middle zone and slightly later than flowering plants at the base zone, suggesting a mismatch. We conclude that, while elevation shapes species distribution, it also differentially influences species phenology. This may be of great significance in long-term assessment of species distribution in sensitive mountain ecosystems.

## Introduction

Mountains are highly significant drivers of species diversity and dispersion patterns. They occur in about half of Earth’s biodiversity hotspots^1^ and are often rich in endemic species diversity^2^. Different elevation zones have different degrees of exposure to weather and climate^3^. Besides the standard lapse rate in temperature, there is great variation in wind, humidity, precipitation, and orographic patterns, differentially affecting the biota at various elevations^4^, especially species abundance and distribution patterns across the elevation gradient^5^.

Species abundance and ecological interaction usually decrease with elevation^6^. However, there is not always a monotonic response in species diversity across elevation gradients. Sometimes, species richness and abundance increase and peak at mid-elevations^7^, then decrease with increasing elevation. However, this response pattern is dependent on taxonomic group. For example, abundance of beetles shows an arched-relationship with elevation^8^, as does that of bumble bees^9^ at mid-elevations. In contrast, army ants show a linear decline in abundance with increasing elevation^10^. According to Rahbek^11^, 50% of studies on species response to elevation gradients show arch-shaped species richness patterns, while species richness with linear patterns make up only 25%. The determining factors of differential response among taxa to elevation gradients are not well understood, although bees track flowering plant diversity^12, 13^, which in addition to abiotic factors, influences bee distribution in terms of species richness and abundance across elevation gradients. Flies on the other hand, are better associated with cold temperatures, and often dominate at high elevations beyond the limits of other taxa^14–16^.

In addition, elevation shapes abundance peaks and species composition^17^. The adaptation of different taxa to differing air temperatures associated with elevation may influence species abundance and composition. For example, flies are well adapted to cool and wet areas, while bees are mostly in dry and warm habitats^18, 19^. In the case of flowering plants, some flowering plant species have low flower abundance associated with increasing spring temperature^20^, especially at low elevations. However, for some mass-flowering species, high spring temperature drives high abundance^21^. In a sensitive ecosystem like the Cape Floristic Region (CFR) of South Africa, where bee diversity matches plant diversity^22^, high plant productivity may ensue when mass flowering is associated with high bee abundance in warm elevation zones. Some studies have shown how bee diversity and composition are driven mostly by the indirect effect of climate change on temporal distribution of floral resources^23–25^, yet there may be differences in species response influencing productivity across elevation zones. For flowering plants at high elevation, the low visitation rate may not always yield poor seed production as the stigmas of some flowering plants at peak elevations are receptive for longer duration^6^, where there is no significant difference in the possibility of plant pollination between high and low elevations.

Factors driving species distribution patterns across elevation gradients also influence their phenology over space and time^26, 27^. Phenology usually describes species’ natural seasonal patterns, which may be associated with seasonal appearance^28, 29^, abundance peaks^16, 30^, flowering duration^31, 32^, and insect flight duration^33, 34^, among other life activities. Shifts in phenology of important flower-visiting insect taxa provide evidence of global change^33, 35^. For example, butterflies are a model taxon for understanding climate change through shifts in phenology^29, 35, 36^. Using temporal patterns of species phenology to understand environmental change is well established^26, 33, 37^. However, studies assessing spatio-temporal response of phenology are few^27, 38, 39^. Although global warming can bring about early onset in appearance of flowering plant and bee species^26, 40^, little information is available on how elevation mediates this pattern.

While most of the spatio-temporal studies addressing insect and plant diversity have been across latitudinal gradients^38^, studies across elevation gradients have only recently been conducted^16, 17^. We investigate this in the CFR, a Mediterranean-type ecosystem with a rich and complex topography across most of its expanse. The mountains are ancient (about 600 my at base to 300 my at summits), and support extensive species radiation of angiosperms, characterized by the sclerophyllous fynbos vegetation^41^. Specifically, we focus on how elevation zones influence species composition and peaks in richness and abundance of different insect pollinator taxa in the CFR. We asked the following: (1) How does elevation affect species abundance and richness of flower-visiting insects, and importantly, how does this differ among taxonomic groups? (2) Are there differences in species assemblages among taxonomic groups of flower-visiting insects in response to elevation shift? (3) Do differences in species assemblages of flower-visiting insects track those of flowering plants with increasing elevation? (4) How does elevation shape species phenology among flower-visiting insect taxonomic groups in terms of abundance peak periods?

## Results

A total of 4 912 insect individuals belonging to 253 morphospecies were sampled (Supplementary Table S1, which includes family names). Beetles constituted 78.5% of the total collection, and far behind, were bees (9.7%), flies (8.9%) and wasps (2.3%). An average of 82%, 75% and 71% Jackknife1 estimated species richness values were recorded for beetles, flies and bees respectively, with wasps (60%) having the lowest completeness (Supplementary Table S2). While the rarefaction curve of beetles across sampling sites approached asymptote (Supplementary Fig. S1), the rarefaction curve of other taxonomic groups across sampling sites did not reach asymptote (Supplementary Fig. S1). A total of 52 flowering plant species were also recorded across all the elevation zones over the sampling period. The rarefaction curve for flowering plants (Chao = 82.30, Jackknife1 = 76.24) across the study sites reached an asymptote (Supplementary Fig. S2).

**Figure 1:**
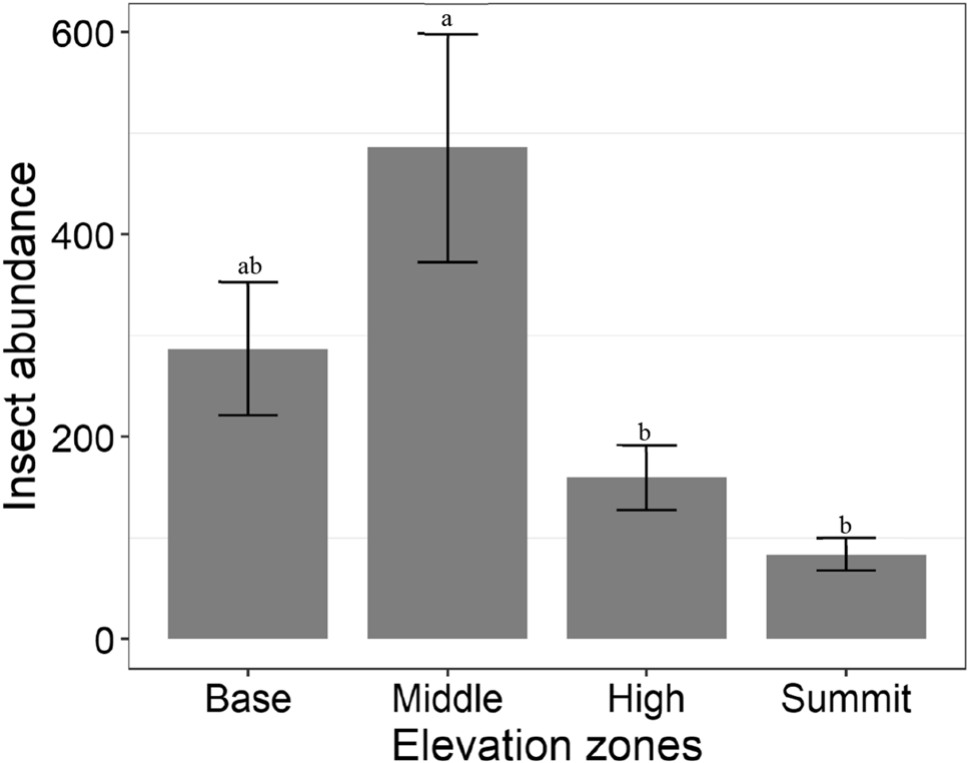
Mean insect abundance (± SE) among elevation zones. Bars with common letters are not significantly different at p > 0.05.

Overall, there was a significant difference in insect abundance across the elevation zones (F_3, 14_ = 5.1, *p* = 0.014). Highest insect abundance was recorded in the middle zone, and lowest in the summit zone (Fig. 1). There was no significant difference in species richness of insects across elevation zones (F_3, 14_ = 1.12, *p* = 0.373).

**Figure 2:**
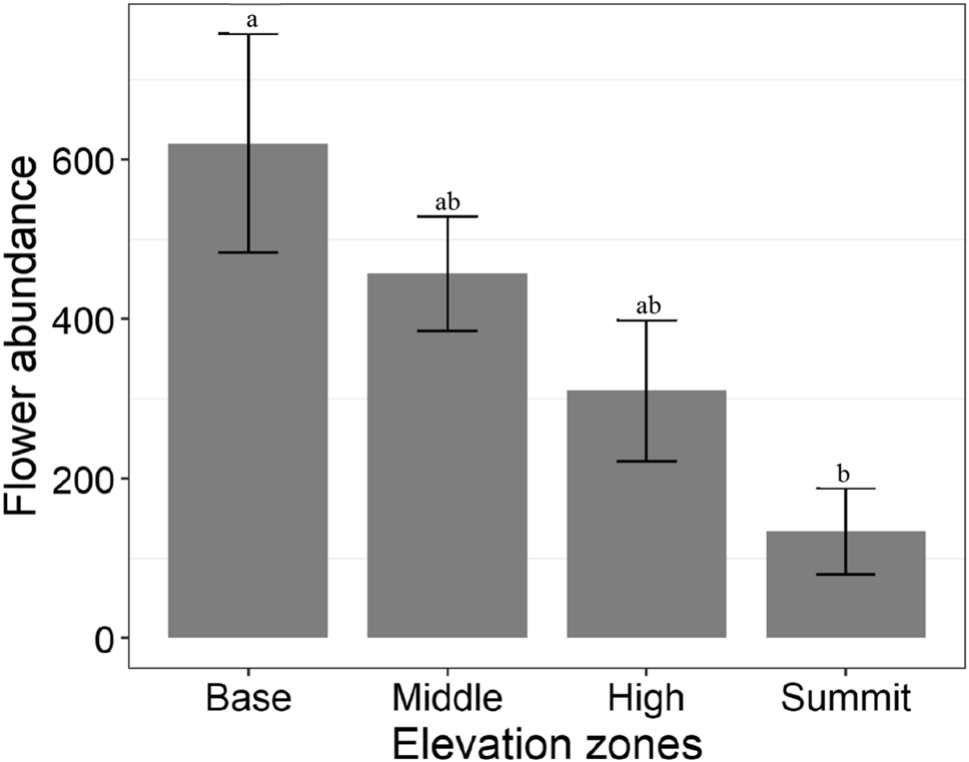
Mean flower abundance (± SE) among elevation zones. Bars with common letters are not significantly different at p > 0.05.

There was a significant difference in flower abundance across elevation zones (F_3, 14_ = 3.637, *p* = 0.039). Highest flower abundance was in the base zone, and lowest in the summit zone (Fig. 2). There was no significant difference in flowering plant species richness among elevation zones (F_3, 14_ = 3.186, *p* = 0.057).

### Abundance and richness among insect taxa

Across elevation, there was no significant difference in the abundance and species richness of bees, flies and wasp. However, beetles were significantly highest in abundance (z = − 2.22, *p* = 0.04) and species richness (z = − 2.52, *p* = 0.02) at the base zone and lowest at the peak elevation.

**Figure 3:**
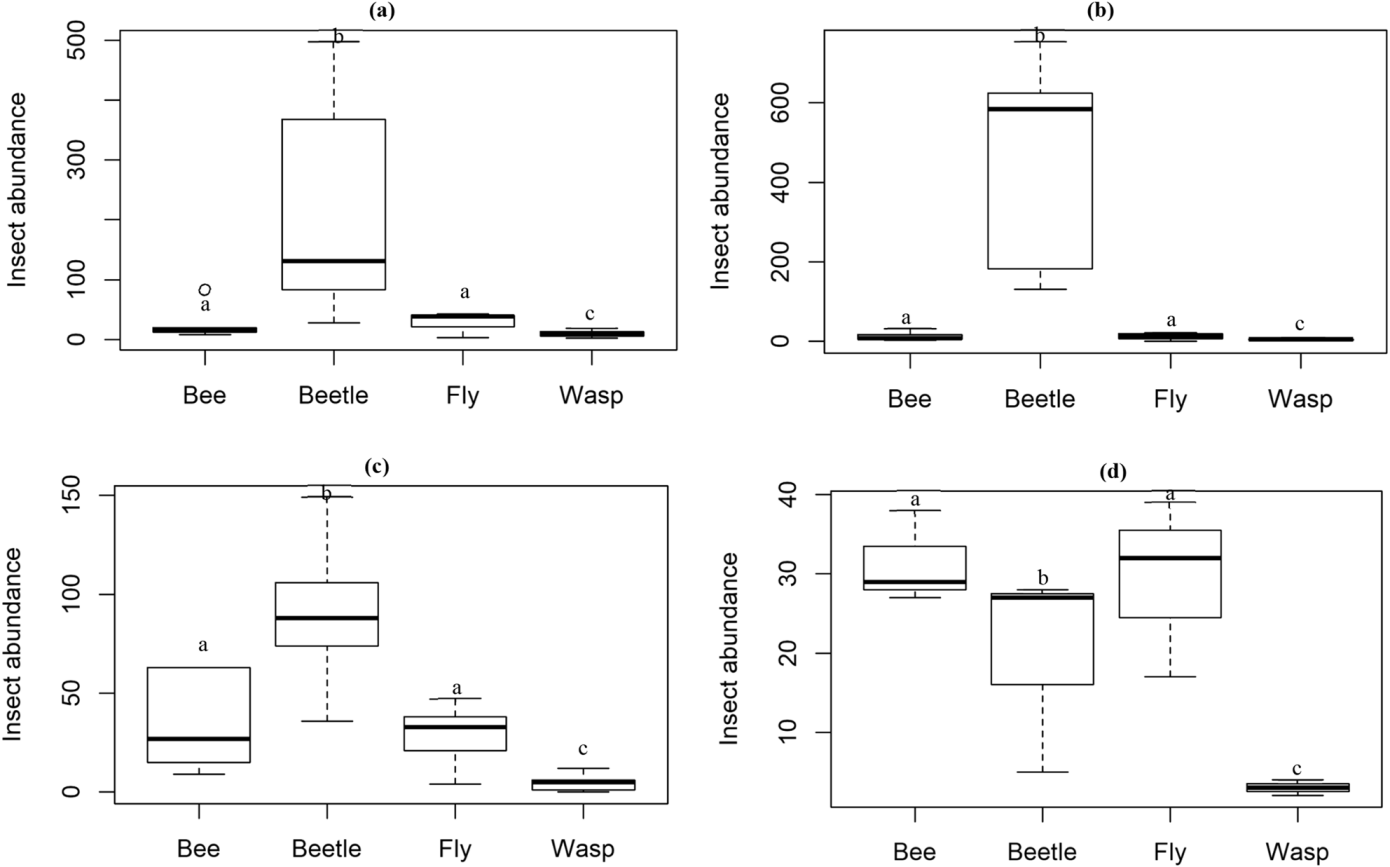
Abundance of flower-visiting insects at the (**a**) base zone, (**b**) middle zone, (**c**) high zone, and (**d**) summit zone among taxonomic groups. Taxa with common letters are not significantly different at p > 0.05.

There was a significant difference in insect abundance among taxonomic groups at the base, middle, high, and summit zones. Beetles made up the dominant group at the three lowest elevations, especially at the middle-elevation zone, but beetle abundance declined sharply at the summit, where bees and flies were more abundant (Fig. 3). Wasp abundance was the lowest among taxonomic groups over all the elevation zones. Across taxonomic groups, beetles were the most abundant, and this was significantly different from the lowest abundance recorded for wasps at the base zone (z = − 21.49, *p* < 0.0001, df = 16), middle zone (z = − 22.90, *p* < 0.0001, df = 16), high zone (z = − 14.03, *p* < 0.0001, df = 16) and summit zone (z = − 5.307, *p* < 0.0001, df = 8).

**Figure 4:**
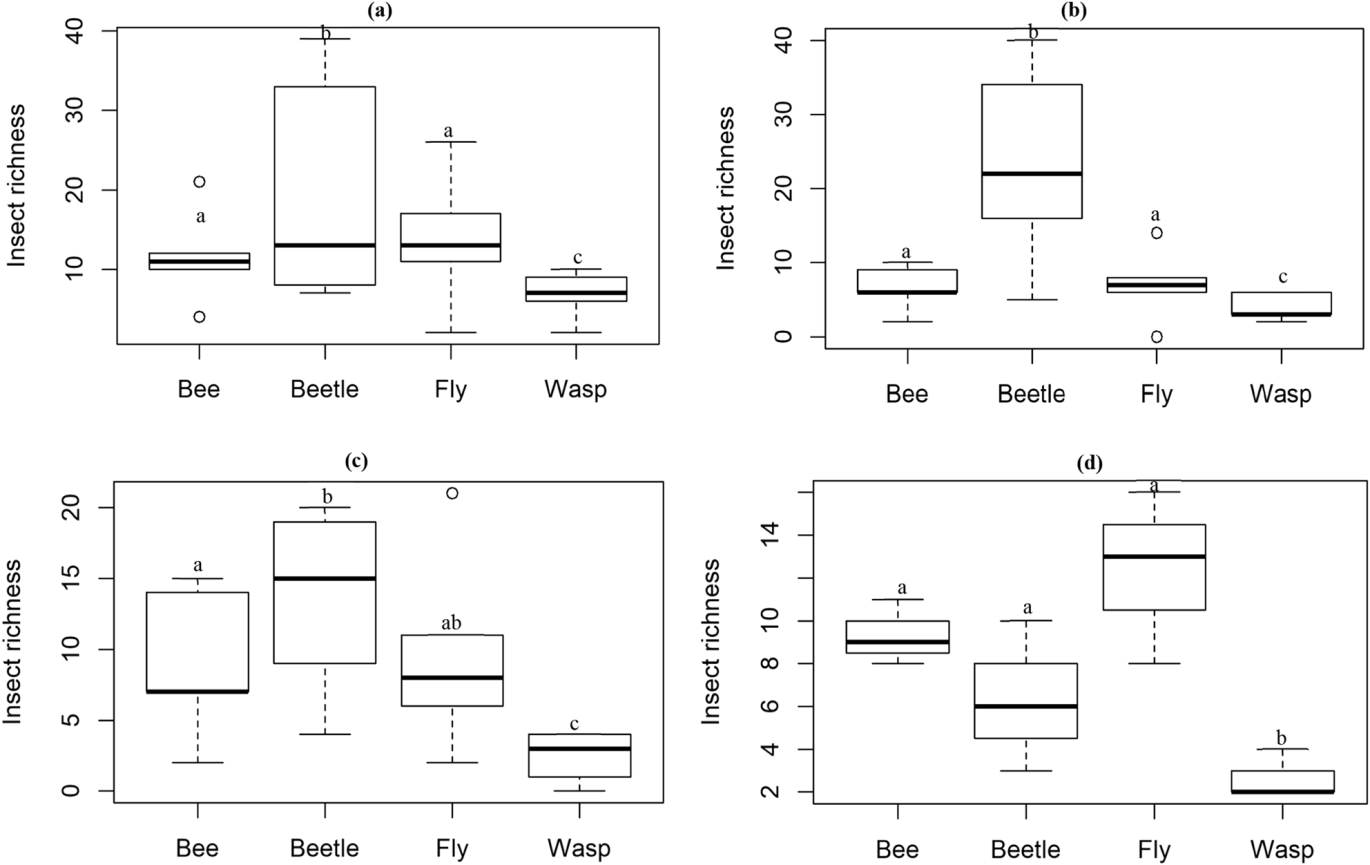
Species richness of flower-visiting insects at the (**a**) base zone, (**b**) middle zone, (**c**) high zone, and (**d**) summit zone among taxonomic groups. Taxa with common letters are not significantly different at p > 0.05.

Similarly, there was a significant difference in insect species richness among taxonomic groups across all elevation zones. Beetles were the most species-rich taxon in the three lowest elevation zones. However, species richness of beetles declined at the summit, where flies and bees showed highest species richness (Fig. 4). Overall, beetles showed the highest species richness among insect taxa, and this was significantly different from the low species richness recorded for wasps at the base zone (z = − 5.434, *p* < 0.0001, df = 16), middle zone (z = − 7.300, *p* < 0.0001, df = 16), high zone (z = − 5.486, *p* < 0.0001, df = 16), and summit zone (z = − 2.052, *p* = 0.04, df = 8).

### Phenology among taxonomic groups

**Figure 5:**
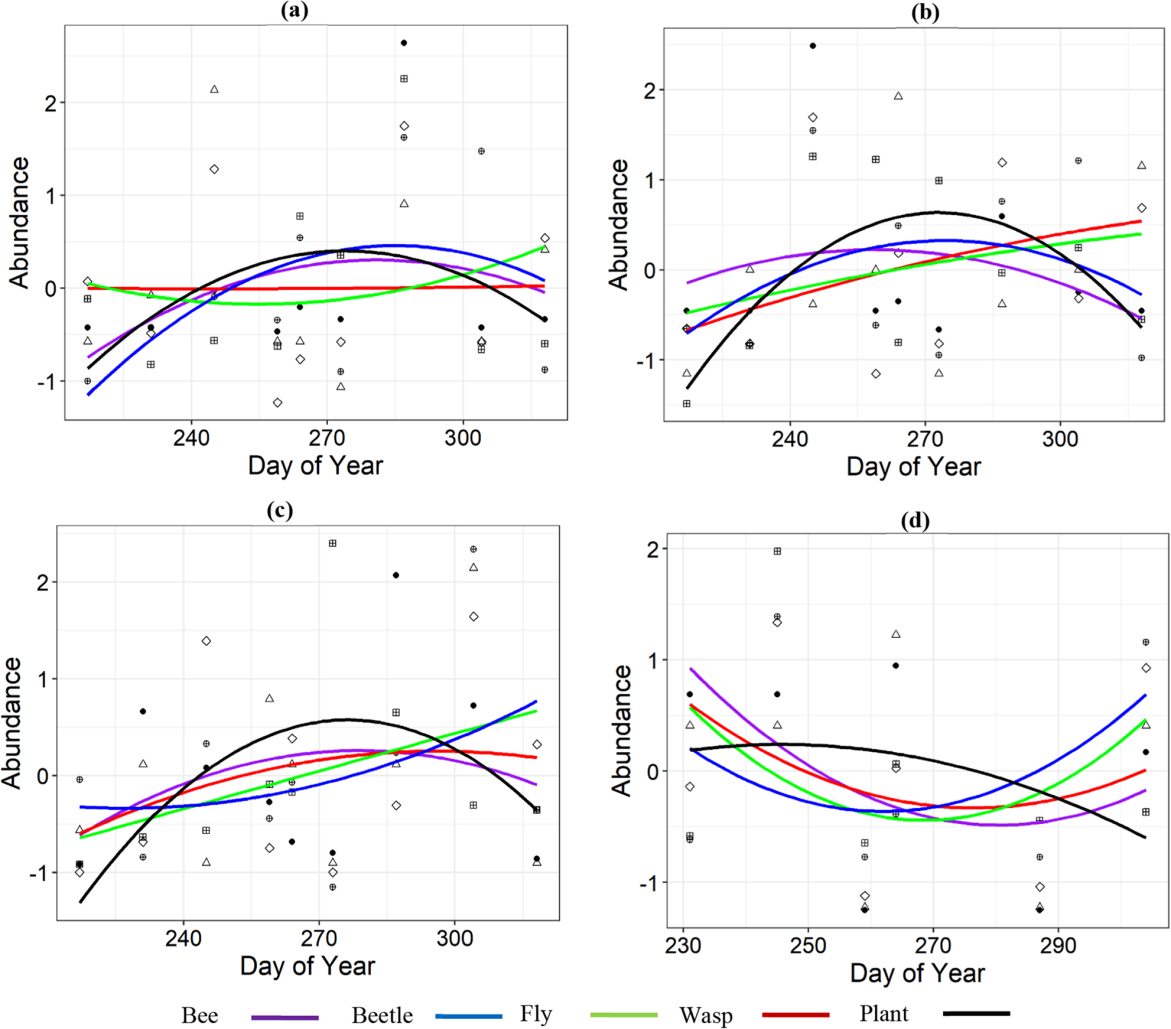
Abundance of insect taxa and flowering plants across sampling days in the (**a**) base zone, (**b**) middle zone, (**c**) high zone, and (**d**) summit zone. Lines represent the best models for and the dots represents abundance recorded at each sampling period.

There was significant difference in species abundance across Days of Year (DOY) and taxonomic groups at the base elevation zone. While bees and beetles showed distinct abundance peaks, flies and wasps showed none. There was no significant difference in abundance among bees and beetles (z = 0.534, *p* = 0.593, df = 35). However, flowering plants peaked ~ 6 days earlier than bees (z = − 2.611, *p* = 0.009, df = 35) and ~ 10 days earlier than beetles (z = − 7.8, *p* < 0.0001, df = 35, Fig. 5a).

Similarly, at the middle-elevation zone, there was a significant difference in species abundance across DOY and taxonomic group. At this zone, there was significant difference in abundance peaks of bees and beetles (z = − 2.096, *p* = 0.036, df = 35), with bees reaching peak abundance 7.5 days before beetles. There was no significant difference in peak abundance of bees and flowering plants (z = − 1.876, *p* = 0.06, df = 35). However, bees peaked in abundance 7.5 days earlier than flowering plants (Fig. 5b).

There was no significant difference in abundance across sampling days and taxonomic group at the high-elevation zone (Fig. 5c). Although at the summit, species abundance changed significantly, a result of the interaction between DOY and insect taxa. None of the insect taxa showed significant peak abundance over the sampling period (Fig. 5d).

### Species composition

**Figure 6:**
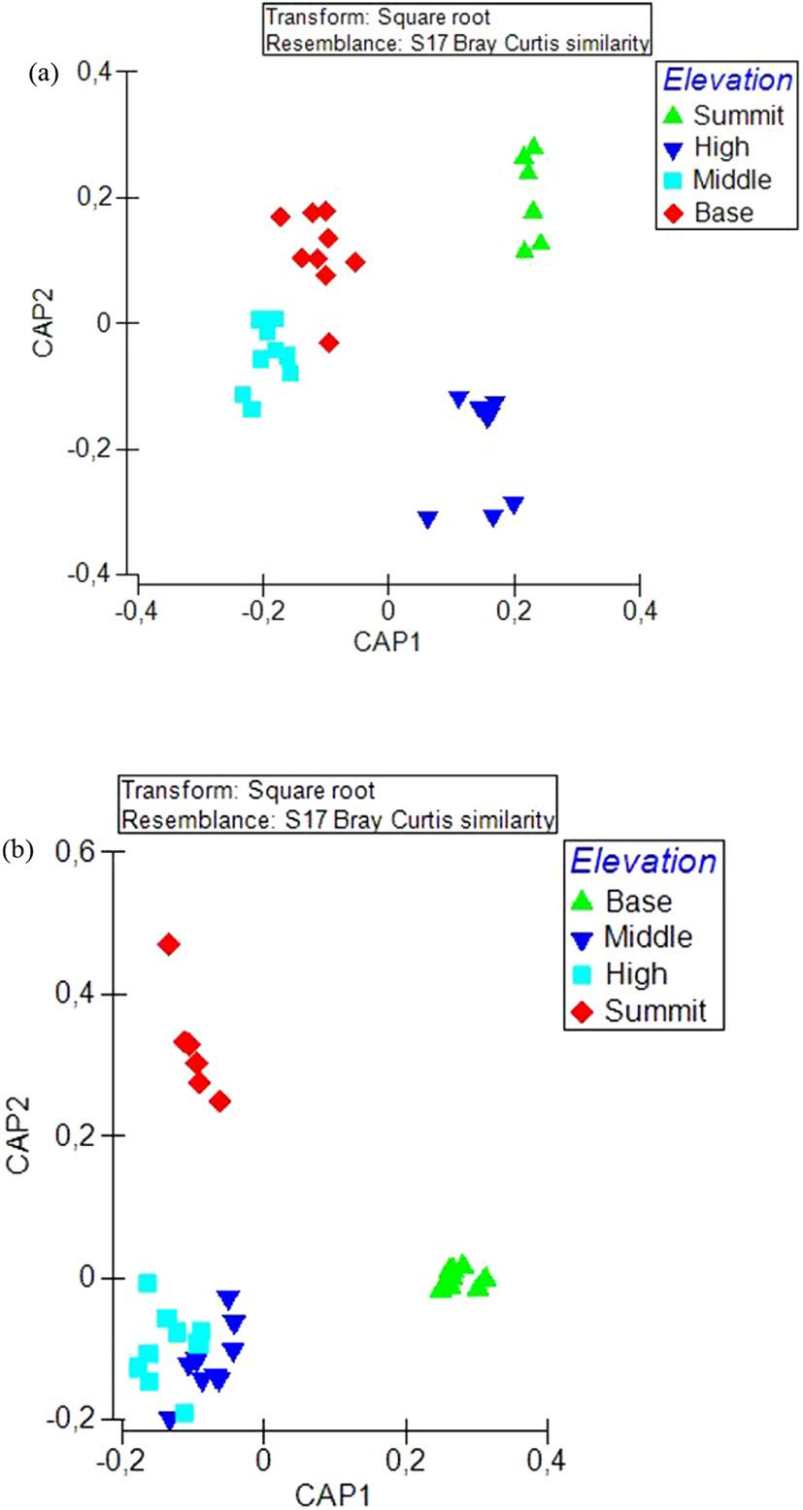
Canonical analysis of principal coordinate showing differences in (**a**) flower visiting insect species composition, and (**b**) flowering plant species composition across elevation zones. CAP plot was computed using Bray-Curtis similarity index obtained from the square-root transformation of abundance data.

There was a significant difference in species composition of insects across elevation zones (Pseudo-*F* = 2.7114, *p* = 0.001). Species composition among any two elevation zones was significantly different. However, the most significant dissimilarity in species composition was among species recorded at the summit and middle elevations, as well as in the summit and base zones (Fig. 6a). Similarly, there was a significant difference in species composition of flowering plants across the elevation zones (Pseudo-*F* = 4.5306, *p* = 0.001). Flowering plant species composition showed strong differences across any two of the elevation zones (Fig. 6b). There was a significant correlation in resemblance matrix of flowering plant composition and flower-visiting insect composition across elevation zones (Rho = 0.138, *p* = 0.0008).

### Species composition among insect taxa

**Figure 7:**
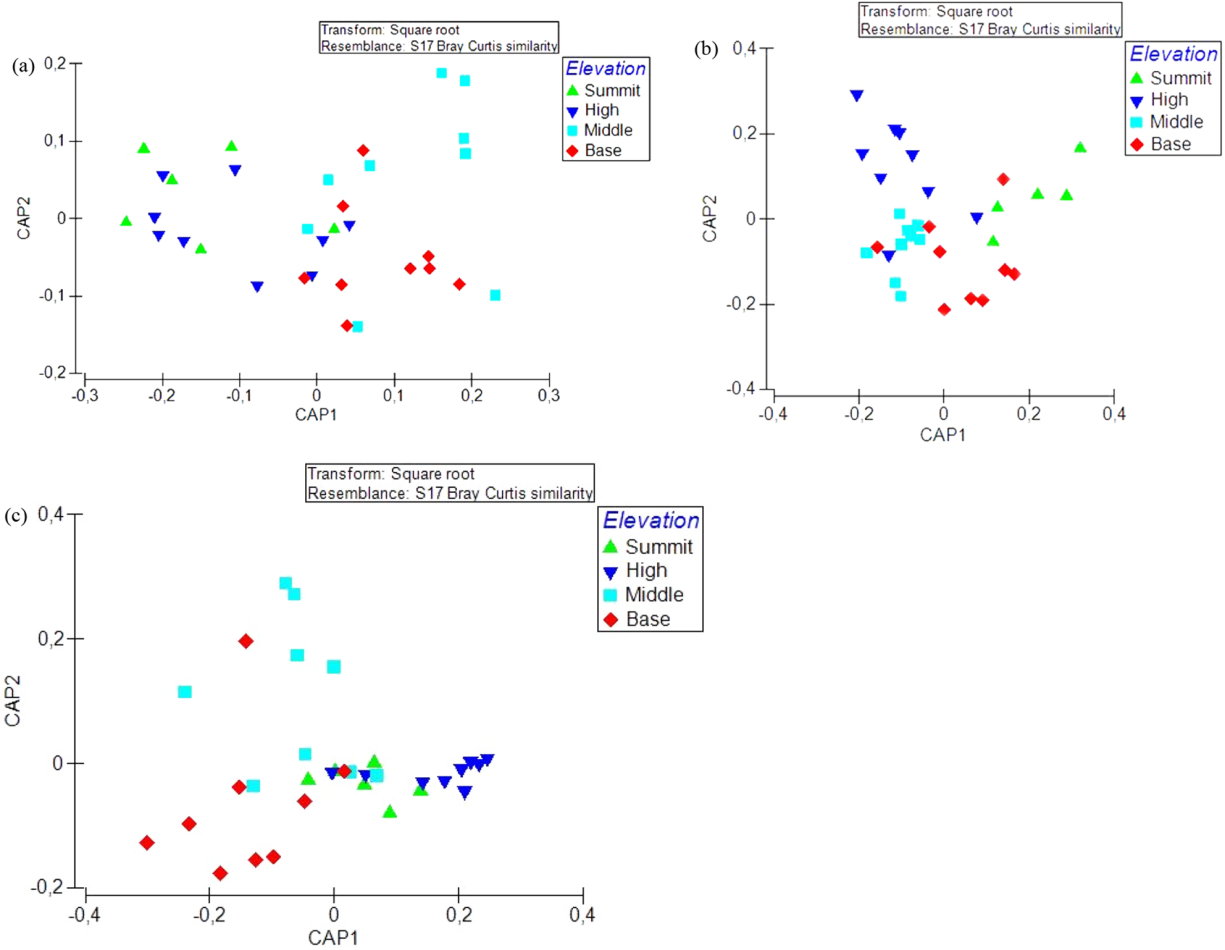
Canonical analysis of principal coordinates, showing differences in species composition of (**a**) bees, (**b**) beetles, and (**c**) wasps across elevation zones. CAP plot was computed using Bray-Curtis similarity index obtained from the square-root transformation of abundance data.

There was a significant difference in species composition of bees across all elevation zones (Pseudo-*F* = 2.268, *p* = 0.003). The most significant difference in species composition was among species in the middle and summit zones, base and summit zones, high and middle zones, as well as high and base zones. There was no significant difference in bee species composition in middle and base zones, and summit and high zones (Fig. 7a).

Similarly, there was a significant difference in species composition of beetles across the elevation zones (Pseudo-*F* = 2.2405, *p* = 0.001), with the strongest difference being between the summit and middle zone. Species among other zones showed weak differences, with no significant difference in species composition in middle and base zones (Fig. 7b).

There was no significant difference in species composition of flies across the elevation zones (Pseudo-*F* = 1.322, *p* = 0.057). There was significant difference in species composition of wasps across the elevation zones (Pseudo-*F* = 4.531, *p* = 0.001). Pairwise comparison showed a significant difference in wasp species in summit and base zones, high and middle elevation zones, high and base zones, and summit and middle zones. However, there was no significant difference in species composition among other zone combinations (Fig. 7c).

## Discussion

### Abundance and species richness

There were distinct differences in elevation response among individual groups of flower-visiting insect taxa and flowering plants. Overall, flower abundance peaked at the middle-elevation zone, with few or no flowers at the summit. Similarly, insects overall showed a mid-elevation peak in abundance, with a major decline above the middle-elevation zone. However, there were large differences among the abundance of the various insect taxa across elevation zones.

Abundance of beetles showed a distinct response to changes in elevation, which is common among this group of anthophilous insects^8^, compared to the relatively stable abundance of bees and flies across elevation zones. While beetle abundance constituted > 70% of total insects collected at the lowest three elevation zones, beetle abundance dropped abruptly at the summit, where there was high abundance of bees and flies, supporting studies that have shown great sensitivity of beetles to changes in environmental variables across elevation gradients^42, 43^. This suggests that beetles are a more suitable indicator of elevation shifts and associated changes in environmental variables than the other three taxa.

Monkey beetles are important pollinators in this region, contributing to agricultural productivity and maintenance of ecosystem functions^44–46^. While the CFR is a diverse and complex topographic landscape, patterns of beetle abundance observed here suggest limited contribution by this group of beetles to pollination of flowering plants at higher elevations. This is a major concern for specialized beetle-pollinated plants at higher elevations. Flies on the other hand are known to be highly abundant at high elevations where other insect taxonomic groups are less represented^14, 16, 47^. In our study, there was no significant difference in the abundance and species richness of flies across elevation zones, this may be attributed to the limited range of our mountain (1,640 asl) compared to other studies that have shown distinct variation in the abundance of flies with elevation^14, 47^. However here, among taxonomic groups, flies and bees were more abundant compared to beetles at the peak elevation. The presence of flies and bees across all elevation zones at relatively equal abundance suggests that these taxa are important pollinators of flowering plants, especially at higher elevations where beetles are absent.

### Phenology among taxonomic groups at elevation zones

Patterns of temporal phenological peaks in abundance among taxonomic groups differed across elevation zones. At the start of sampling, there was snow in the summit zone, which only began to warm up towards the middle of the sampling season. Steady increase in abundance and richness of insects began towards the end of the sampling season, especially for flies and bees in the summit zone. This likely explains why insects showed no distinct abundance peak period across sampling time compared to other elevation zones.

Bee abundance showed a clear early peak compared to the other taxonomic groups at all elevations. This is important for the pollination of plants that flower early in the season, as interactions between the pollinators and flowering plants were previously recorded in the same study area as here^48^. Compared to bees, wasps showed no distinct peaks in abundance across elevation zones, except the high zone. One possible explanation is differences in ecosystem functional role of each taxon, which may shape species distribution across these zones^49^. While most adult wasps feed on nectar and can act as pollinators, they can also act as predators, parasitoids, or even parasites, especially the larval stages utilizing ecosystem components differently. For example, most female parasitoid wasps will feed on the hemolymph of their hosts as additional source of nutrient for egg production^50^. Unlike bees, which are obligate florivores, the distribution of wasps across elevation may be influenced by the availability of other resources. Also, wasps are mostly generalists, especially at high elevations where they share floral resources with flies^51^. It is unclear what drove the lack of distinct wasp abundance peaks across elevation zones here. However, while other groups are expected to respond to peak flowering spring season, long-term studies across all seasons might better reflect patterns of wasp abundance distribution across elevation zones.

Beetles peaked in abundance later in the season than bees at all elevation zones. Beetles are sensitive to temperature changes^42^, and while bees and flies forage around flowers, monkey beetles here were mostly in the cone of *P. repense* flowers, especially in the high zone. In addition, bees and flies are strongly associated with flower abundance, as most are nectar feeders. However, monkey beetles eat mostly pollen, and sometimes bore into cavities of flowers where they also seek shelter^52^. Overall, species belonging to different taxonomic groups may respond at different rate to environmental conditions that are distributed differentially across elevation gradients^53^.

Bees are the most essential pollinators in several terrestrial ecosystems, and a delay in their appearance may result in a mismatch with the phenology of flowering plants, leading to poor pollination^34, 54, 55^. Here we found asynchrony in abundance peaks of bees and flowering plants at the base and middle-elevation zones. Of concern is the conflicting pattern of difference, where bees peaked earlier than flowering plants at the middle-elevation zone, but later than flowering plants at the base. While short-term observations may not affirm the mismatch observed here, such differences in abundance peak period of bees and angiosperms can result in poor productivity of flowering plants, especially when the plants are dependent on bees for pollination^56^, as in the case of species-rich Asteraceae at the middle and base elevation zones^48^. Although the mismatch observed at the middle zone was not statistically significant, a distinct early peak in abundance of bees was observed there. Long-term monitoring of plant and bee phenology on our mountain may establish the pattern observed as contrasting abundance peaks of flowering plants and bees has been associated with climate change^57, 58^. Mostly, long-term phenological data have shown insects in general to be more sensitive to springtime temperature rise compared to flowering plants per se^55, 57, 59^. While this may be the plausible explanation for the results here, the patterns may also be influenced by species seasonal flight duration, which can contrast with the flowering peak period^34^.

### Differences in species composition

Species composition in all four groups of flower-visiting insects differed significantly across elevation zones. Species in the summit zone segregated distinctly from species in the middle and base zones. Similarly, there was a distinct segregation of species composition of flowering plants across all elevations. Each of the zones is characterized by a distinct vegetation type^60^, suggesting some isolation among elevation zones in terms of flowering plant species. The summit zone was characterized by dwarf mountain fynbos plants (mostly restios and ericas), most of which are wind pollinated, except for a few insect-pollinated flowering plants restricted to this zone. This shows some plants can be successfully pollinated even in the absence of insect pollinators, which are not abundant at this zone.

The resemblance matrix of insect composition correlated significantly with the resemblance matrix of the flowering plants. Although habitat size, in terms of flower abundance, influenced abundance of flower-visiting insects, habitat quality in terms of flower composition was equally essential, as it is associated with the distribution of flower-visiting insect species across the elevation zones. Here, functional diversity of plant and pollinators may explain the correlation between insect and flowering plant species composition. Elevation predicts functional diversity of flowering plants^61^ and this may also attract different subsets of pollinators at different elevation zones. Thus, at higher elevation zones, and where abiotic factors are extreme, the presence of flowering plants and pollinators that are adapted to conditions at these zones may enhance stability of ecosystem functions, even in the peak zone.

According to Agenbag^60^ and Adedoja^48^, the lowest three elevation zones have some common plants species including *P. repense, Metalasia muricata, Lobostemon* sp.*, Senecio* sp.1.*, Senecio* sp.2.*, Muraltia* sp*., Cullumia* sp., etc. However, in contrast, species in the summit zone, and which also occurred elsewhere on this mountain, displayed dwarfism such as the invasive *Acacia* sp., and *Protea repense*, which was common in both the summit and other elevation zones. While the lowest three elevation zones are more connected in terms of flowering plant species composition, the peak zone here, although having low plant richness, is distinct in terms of unique plant species. This shows the importance of conserving the limited but uniquely adapted plant species here, which might otherwise be prone to temporal loss and local extinction on this mountain.

## Conclusion

While it is important to monitor species’ temporal shifts in response to climate change, elevation shifts may shape this response among various taxa, as seen here. Other studies have shown how insect and flowering plant phenology changes across temporal scales in the context of climate change. Here, our results show how zonation across elevation gradients differentially influences the abundance peak period of different taxonomic insect groups, and relative to that of flowering plants.

The results here are important for global studies addressing how species phenology is affected by climate change over time. There may be large fluctuations across years of climate monitoring. However, the real-time changes across elevation zones may be of great significance. While some studies have shown temporal changes in species phenology at common elevations^33, 37^, monitoring across elevation gradients over time may show some interesting patterns. Importantly, in-depth studies of the impact of climate change on biodiversity should also take into account the pattern of species phenology across elevation zones.

## Sites and methods

The study was undertaken on Jonaskop Mountain (33° 58′ 10.67″ S, 19° 30′ 21.96″ E), Western Cape Province, South Africa, in the Cape Floristic Region biodiversity hotspot. The bee diversity of the CFR is exceptionally high, coinciding with that of plants^22^. Jonaskop Mountain, our focal study area, reaches 1,640 m a.s.l., and supports many localized sclerophyllous fynbos plant species. The mountain is highly exposed to extreme weather events (cold, wet and windy in winter, and hot and dry in summer), has distinct vegetation zones^51^, and is a sentinel mountain for monitoring climate change.

Our study sites, with increasing elevation on the mountain, are those used previously in vegetation profiling^60^. These sites were also previously surveyed for plant-pollinator interactions in 2017^48^, including the flowering plants and insects studied here. Low elevations (< 550 m a.s.l., 33° 55′ 03.8″ S, 19° 30′ 46.1″ E, ‘Base zone’) are characterized by succulent karoo. Elevations 650–744 m a.s.l. (33° 55′ 28.2″ S, 19° 30′ 59.4″ E, ‘Middle zone’) are an ecotone between the lower elevations, and the third zone (33° 57′ 06.5″ S, 19° 31′ 02.0″ E ‘High zone’) and characterized by Mid-elevation Sandstone Fynbos at 953–1,303 m a.s.l. The highest elevation (> 1576 m a.s.l., 33° 58′ 09.0″ S, 19° 29′ 45.3″ E, ‘Summit zone’) is classified as High-elevation Sandstone Dwarf Fynbos^62^.

Flower-visiting insects in the area belong mostly to four major taxa: bees and wasps (Hymenoptera), beetles (Coleoptera: mostly Scarabaeidae, as well as some beetle families whose roles as flower visitors have been identified from literature), and flies (Diptera: mostly Syrphidae, Bombyliidae and Muscidae, including other families whose role in flower visitation has been recorded). These taxa were sampled across 18 study sites between August-November 2017 and 2018, the flowering time of most plants in the area. Five study sites each were established in the first three zones with only three study sites established at the summit zone due to shortage of suitable sites. Sites were selected with increasing elevation from 385 m–550 m a.s.l. (Base zone), 712–740 m a.s.l. (Middle zone), 990 m–1,250 m a.s.l. (High zone) and > 1,570 m a.s.l. (Summit zone). Sites were selected along increasing elevation at each zone based on their abundant flower cover.

Each site was a 50 m^2^ plot. Plots within any one zone were 100–500 m apart. Groups of these sites, representing the four zones, were 0.8–2 km apart. Nine visits were made to each of the lowest three zones. Each site was visited twice (once in each year) with the exception of one site in each of the middle, high and base zones which were partly or completely burned in the second year. Six visits were made to the summit zone with every site visited in the second year. These were the only areas with flowers in the summit zone. Pooled insect and plant abundance data in each site were used to assess differences in species abundance across elevation zones. However, repeated collections in different sampling days in the same sites gave an assessment of species phenology across sampling days and elevation zones.

Insects were sampled with yellow pan traps at all elevation zones. White and blue pan traps were included in a pilot study. However, these collected very few insects and so were not used. At each collection time, 10 yellow bowls (2 L) were half-filled with water, and raised to vegetation height at each elevation zone for a period of 24 h. Insects caught in each bowl were retained, and stored in 70% ethanol. They were then assigned to morphospecies with reference to collections from this region and using appropriate taxonomic guides^63–65^. Rare/new species (mostly singletons) were identified as a new species/morphospecies by expert taxonomists specializing in different insect taxa in the Department of Conservation Ecology and Entomology, Stellenbosch University. Morphospecies counts of individuals were used as an approximate abundance measure in the statistical analyses.

Flower abundance was estimated at each study site in five replicates of 2 m^2^ plots. A flower unit was defined here as the unit from which a honeybee-sized insect will fly to the next unit rather than walk^66^. Flower units of identified plant species in each plot were counted, and data pooled over the entire 50 m^2^ plot. Similarly, flowering plant species richness was estimated at each study plot across all elevation zones at each visit.

### Statistical analyses

To assess sampling adequacy, we estimated species rarefaction curves for flowering plants and each flower-visiting insects taxa species richness across study sites using the *rarefy* function in *vegan* R package^67^. We also used the Chao1, Jackknife1 and ICE (Incidence based estimate) estimators based on abundance data to quantify sampling completeness. We further computed the percentage species richness for each taxon according to sampling completeness using the observed species richness and Jackknife1 estimate of each taxon.

Analysis of variance (ANOVA) was used to test for difference in overall insect abundance and species richness across elevation zones and insect taxonomic groups. Pooled data for abundance and species richness of insects and flowering plants across site visits were normally distributed according to Shapiro–Wilk’s normality test. Tukey HSD post-hoc test was computed to observe pairwise comparison among groups. To assess how insect abundance and species richness differed among taxonomic groups at each elevation, we used a generalised linear model (GLM) in R version 3.4.1^68^ and specifying Poisson distribution. Here, abundance and species richness were specified as dependent variables in different models and insect taxa at each elevation included as the fixed factor.

To investigate how species composition varied across elevation gradient, we used a Bray Curtis dissimilarity matrix, and compared this among elevation zones using permutation multivariate analysis of variance (PERMANOVA) in Primer 6 software^69^. The PERMANOVA was performed in both cases using the Bray–Curtis similarity index obtained from the square-root transformation of abundance data and selecting Type III SS sum of square to account for unbalanced design^70^. Data were permuted 999 times for the analysis.

We further tested for pairwise comparisons where significant differences were observed. Similarly, the same pattern was applied to assess the difference in the species composition of flowering plants across elevation zones using square-root transformation of abundance data. To understand whether insect composition tracks flower composition, we compared the resemblance matrix of flower-visiting insects with that of flowering plants across elevation zones using the RELATE function in Primer 6 software.

To determine the period of peak abundance during the sampling season and differences among taxonomic groups across sampling days (using abundance data collected in each site visit), we converted sampling days to respective Days of the Year (DOY) (continuous numerical values of 1–366 (leap year) across the calendar year) for each elevation zone^16^. GLMs (specifying Poisson distribution) were used to assess the relationship between abundance and DOY of sampling. To establish the abundance peak at each zone among insect taxa, insect abundance was specified as the dependent variable. Day of Year and insect taxonomic group at each elevation zone were the fixed factors. We also included the quadratic term of Day of Year (DOY^2^, and we observed two-way interactions between DOY and each insect taxon, and between DOY^2^ and each insect taxon. We also computed this using a stepwise model simplification, while removing non-significant variables or interactions.

## Supplementary information

Supplementary Information 1.

Supplementary Figure S1.

Supplementary Figure S2.

## Data Availability

Data will be submitted to Dryad online repository upon the acceptance of the manuscript by the journal.
