# Supplementary material for: Asynchrony among insect pollinator groups and flowering plants with elevation
Source: Sci Rep. 2020 Aug 6;10:13268. doi: 10.1038/s41598-020-70055-5 (PMC7411018; doi:10.1038/s41598-020-70055-5)
Supplement: Supplementary file 1 — Supplementary Information 1. [file 41598_2020_70055_MOESM1_ESM.docx]

**Asynchrony among insect pollinator groups and flowering plants with elevation**

***Opeyemi Adedoja^1,2^, Temitope Kehinde^3^, Michael J. Samways^1^**

^1^ Department of Conservation Ecology and Entomology, Stellenbosch University, South Africa

^2^Department of Conservation and Marine Sciences, Cape Peninsula University of Technology, South Africa

^3^Department of Zoology, Obafemi Awolowo University, Ile-Ife, Nigeria

Supplementary Table S1. List of anthophilous insect species

| Taxa | Genus | Species/Morphospecies | Family |
| --- | --- | --- | --- |
| Beetles | *Amblymelauoplia* | sp.1 | Scarabaeidae |
| Beetles | *Anaspis* | sp. | Scraptiidae |
| Beetles | *Anisonyx* | *Proletorius* | Scarabaeidae |
| Beetles | *Anisonyx* | sp. | Scarabaeidae |
| Beetles | *Anisonyx* | *ursus* | Scarabaeidae |
| Beetles | *Anthrenus* | *verbasci* | Dermestidae |
| Beetles | *Apalochrus* | sp. | Melyridae |
| Beetles | Buprestidae | sp.1 | Buprestidae |
| Beetles | Buprestidae | sp.2 | Buprestidae |
| Beetles | Buprestidae | sp.3 | Buprestidae |
| Beetles | Buprestidae | sp.4 | Buprestidae |
| Beetles | Buprestidae | sp.5 | Buprestidae |
| Beetles | Buprestidae | sp.6 | Buprestidae |
| Beetles | Buprestidae | sp.7 | Buprestidae |
| Beetles | Buprestidae | sp.8 | Buprestidae |
| Beetles | Buprestidae | sp.9 | Buprestidae |
| Beetles | Buprestidae | sp.10 | Buprestidae |
| Beetles | Cantharidae | sp. | Cantharidae |
| Beetles | Carabidae | sp. | Carabidae |
| Beetles | *Ceroctis* | *capensis* | Meloidae |
| Beetles | Chrysomelidae | sp.1 | Chrysomelidae |
| Beetles | Chrysomelidae | sp.2 | Chrysomelidae |
| Beetles | Chrysomelidae | sp.3 | Chrysomelidae |
| Beetles | Cleridae | sp.1 | Cleridae |
| Beetles | Cleridae | sp.2 | Cleridae |
| Beetles | Cleridae | sp.3 | Cleridae |
| Beetles | Cleridae | sp.4 | Cleridae |
| Beetles | Cleridae | sp.5 | Cleridae |
| Beetles | Cleridae | sp.6 | Cleridae |
| Beetles | *Cloniocerus* | *kraussi* | Cerambycidae |
| Beetles | Coccinellidae | sp. | Coccinellidae |
| Beetles | Dermestidae | sp.1 | Dermestidae |
| Beetles | Dermestidae | sp.2 | Dermestidae |
| Beetles | *Heterochelus* | sp. | Scarabaeidae |
| Beetles | *Hippodamia* | *variegata* | Coccinellidae |
| Beetles | Hoplinii | sp.1 | Scarabaeidae |
| Beetles | Hoplinii | sp.2 | Scarabaeidae |
| Beetles | Hoplinii | sp.3 | Scarabaeidae |
| Beetles | Hoplinii | sp.4 | Scarabaeidae |
| Beetles | Hoplinii | sp.5 | Scarabaeidae |
| Beetles | Hoplinii | sp.6 | Scarabaeidae |
| Beetles | Hoplinii | sp.7 | Scarabaeidae |
| Beetles | Hoplinii | sp.8 | Scarabaeidae |
| Beetles | Hoplinii | sp.9 | Scarabaeidae |
| Beetles | Hoplinii | sp.10 | Scarabaeidae |
| Beetles | Hoplinii | sp.11 | Scarabaeidae |
| Beetles | Hoplinii | sp.12 | Scarabaeidae |
| Beetles | Hoplinii | sp.13 | Scarabaeidae |
| Beetles | Hoplinii | sp.14 | Scarabaeidae |
| Beetles | Hoplinii | sp.15 | Scarabaeidae |
| Beetles | Hoplinii | sp.15 | Scarabaeidae |
| Beetles | *Lepitrix* | *dichropus* | Scarabaeidae |
| Beetles | *Lepitrix* | sp.1 | Scarabaeidae |
| Beetles | *Leucocelis* | *amethysina* | Cetoniinae |
| Beetles | Meloidae | sp.1 | Meloidae |
| Beetles | Meloidae | sp.2 | Meloidae |
| Beetles | Meloidae | sp.3 | Meloidae |
| Beetles | Meloidae | sp.4 | Meloidae |
| Beetles | Meloidae | sp.5 | Meloidae |
| Beetles | *Melyris* | sp.1 | Melyridae |
| Beetles | *Melyris* | sp.2 | Melyridae |
| Beetles | *Neoeutrapela* | sp. | Tenebrionidae |
| Beetles | Nitidulidae | sp.1 | Nitidulidae |
| Beetles | Nitidulidae | sp.2 | Nitidulidae |
| Beetles | Nitidulidae | sp.3 | Nitidulidae |
| Beetles | Nitidulidae | sp.4 | Nitidulidae |
| Beetles | *Peritrichia* | *nigrita* | Scarabaeidae |
| Beetles | *Peritrichia* | sp.1 | Scarabaeidae |
| Beetles | *Promeces* | *longipes* | Cerambycidae |
| Beetles | Rutelinae | sp. | Scarbaeidae |
| Beetles | *Trichostetha* | *fascicularis* | Cetoniinae |
| Beetles | *Trichostetha* | *capensis* | Cetoniinae |
| Bees | *Allodape* | sp. | Apidae |
| Bees | *Allodapula* | sp.1 | Apidae |
| Bees | *Andrena* | sp.1 | Andrenidae |
| Bees | *Anthidiini(Plesiamchidium)* | sp.1 | Megachilidae |
| Bees | *Anthidiini* | sp.2 | Megachilidae |
| Bees | *Anthidioma* | sp.1 | Megachilidae |
| Bees | *Anthidioma* | sp.2 | Megachilidae |
| Bees | *Anthophora* | sp.1 | Apidae |
| Bees | *Anthophora* | sp.2 | Apidae |
| Bees | *Anthophora* | sp.3 | Apidae |
| Bees | *Anthophora* | sp.4 | Apidae |
| Bees | *Anthophora* | sp.5 | Apidae |
| Bees | *Anthophora* | sp.6 | Apidae |
| Bees | *Anthophora* | sp.7 | Apidae |
| Bees | *Apis* | *mellifera* | Apidae |
| Bees | *Brausapis* | sp. | Apidae |
| Bees | *Lasioglossum* | sp.1 | Halictidae |
| Bees | *Lasioglossum* | sp.11 | Halictidae |
| Bees | *Lasioglossum* | sp.12 | Halictidae |
| Bees | *Lasioglossum* | sp.14 | Halictidae |
| Bees | *Lasioglossum* | sp.10 | Halictidae |
| Bees | *Lasioglossum* | sp.8 | Halictidae |
| Bees | *Lasioglossum* | sp.2 | Halictidae |
| Bees | *Lasioglossum* | sp.3 | Halictidae |
| Bees | *Lasioglossum* | sp.4 | Halictidae |
| Bees | *Lasioglossum* | sp.5 | Halictidae |
| Bees | *Lasioglossum* | sp.13 | Halictidae |
| Bees | *Lasioglossum* | sp.15 | Halictidae |
| Bees | *Lasioglossum* | sp.6 | Halictidae |
| Bees | *Lasioglossum* | sp.7 | Halictidae |
| Bees | *Lasioglossum* | sp.16 | Halictidae |
| Bees | *Lasioglossum* | sp.9 | Halictidae |
| Bees | Megachilidae | sp. | Megachilidae |
| Bees | *Melitta* | sp. | Melittidae |
| Bees | Osmiini | sp.4 | Megachilidae |
| Bees | Osmiini | sp.1 | Megachilidae |
| Bees | Osmiini | sp.2 | Megachilidae |
| Bees | Osmiini | sp.3 | Megachilidae |
| Bees | *Pachymelus* | sp. | Apidae |
| Bees | *Patellapis* | sp. | Halictidae |
| Bees | *Plesianthidium* | sp.1 | Megachilidae |
| Bees | *Plesianthidium* | sp.2 | Megachilidae |
| Bees | *Pseudoanthidium* | sp. | Megachilidae |
| Bees | *Redivivoides* | sp. | Melittidae |
| Bees | *Seladonia* | sp.1 | Halictidae |
| Bees | *Seladonia* | sp.2 | Halictidae |
| Bees | *Seladonia* | sp.3 | Halictidae |
| Bees | *Seladonia* | sp.4 | Halictidae |
| Bees | *Seladonia* | sp.5 | Halictidae |
| Bees | *Tetraloniella* | sp.1 | Apidae |
| Bees | *Tetraloniella* | sp.2 | Apidae |
| Bees | *Xylocopa* | sp. | Apidae |
| Wasp | Bethylidae | sp.1 | Bethylidae |
| Wasp | Bethylidae | sp.2 | Bethylidae |
| Wasp | Bethylidae | sp.3 | Bethylidae |
| Wasp | Braconidae | sp.1 | Braconidae |
| Wasp | Braconidae | sp.2 | Braconidae |
| Wasp | Braconidae | sp.3 | Braconidae |
| Wasp | Braconidae | sp.4 | Braconidae |
| Wasp | Braconidae | sp.5 | Braconidae |
| Wasp | Braconidae | sp.6 | Braconidae |
| Wasp | Braconidae | sp.7 | Braconidae |
| Wasp | Braconidae | sp.8 | Braconidae |
| Wasp | Chrysididae | sp.1 | Chrysididae |
| Wasp | Chrysididae | sp.2 | Chrysididae |
| Wasp | Eumeninae | sp.1 | Vespidae |
| Wasp | Eumeninae | sp.2 | Vespidae |
| Wasp | Ichneumonidae | sp.1 | Ichneumonidae |
| Wasp | Ichneumonidae | sp.2 | Ichneumonidae |
| Wasp | Ichneumonidae | sp.3 | Ichneumonidae |
| Wasp | Ichneumonidae | sp.4 | Ichneumonidae |
| Wasp | Ichneumonidae | sp.5 | Ichneumonidae |
| Wasp | Ichneumonidae | sp.6 | Ichneumonidae |
| Wasp | Ichneumonidae | sp.7 | Ichneumonidae |
| Wasp | Ichneumonidae | sp.8 | Ichneumonidae |
| Wasp | Masarinae | sp. | Vespidae |
| Wasp | Pompilidae | sp.1 | Pompilidae |
| Wasp | Pompilidae | sp.2 | Pompilidae |
| Wasp | Pompilidae | sp.3 | Pompilidae |
| Wasp | Pompilidae | sp.4 | Pompilidae |
| Wasp | Sphecidae | sp.1 | Sphecidae |
| Wasp | Sphecidae | sp.2 | Sphecidae |
| Wasp | Sphecidae | sp.3 | Sphecidae |
| Wasp | Sphecidae | sp.4 | Sphecidae |
| Wasp | Sphecidae | sp.5 | Sphecidae |
| Wasp | Sphecidae | sp.6 | Sphecidae |
| Wasp | Sphecidae | sp.7 | Sphecidae |
| Wasp | Sphecidae | sp.8 | Sphecidae |
| Wasp | Sphecidae | sp.9 | Sphecidae |
| Wasp | Sphecidae | sp.10 | Sphecidae |
| Wasp | Sphecidae | sp.11 | Sphecidae |
| Wasp | Sphecidae | sp.12 | Sphecidae |
| Wasp | Sphecidae | sp.13 | Sphecidae |
| Wasp | Sphecidae | sp.14 | Sphecidae |
| Wasp | Sphecidae | sp.15 | Sphecidae |
| Wasp | Sphecidae | sp.16 | Sphecidae |
| Wasp | Vespidae | sp.1 | Vespidae |
| Wasp | Vespidae | sp.2 | Vespidae |
| Wasp | Vespidae | sp.3 | Vespidae |
| Fly | Asilidae | sp.1 | Asilidae |
| Fly | Asilidae | sp.1 | Asilidae |
| Fly | Bombyliidae | sp.1 | Bombyliidae |
| Fly | Bombyliidae | sp.2 | Bombyliidae |
| Fly | Bombyliidae | sp.3 | Bombyliidae |
| Fly | Bombyliidae | sp.4 | Bombyliidae |
| Fly | Bombyliidae | sp.5 | Bombyliidae |
| Fly | Bombyliidae | sp.6 | Bombyliidae |
| Fly | Bombyliidae | sp.7 | Bombyliidae |
| Fly | Bombyliidae | sp.8 | Bombyliidae |
| Fly | Bombyliidae | sp.9 | Bombyliidae |
| Fly | Bombyliidae | sp.10 | Bombyliidae |
| Fly | Bombyliidae | sp.11 | Bombyliidae |
| Fly | Bombyliidae | sp.12 | Bombyliidae |
| Fly | Bombyliidae | sp.13 | Bombyliidae |
| Fly | Bombyliidae | sp.14 | Bombyliidae |
| Fly | Calliphora | sp. | Calliphoridae |
| Fly | Calliphoridae | sp.1 | Calliphoridae |
| Fly | Calliphoridae | sp.2 | Calliphoridae |
| Fly | Calliphoridae | sp.3 | Calliphoridae |
| Fly | Calliphoridae | sp.4 | Calliphoridae |
| Fly | Culicidae | sp.1 | Culicidae |
| Fly | Culicidae | sp.2 | Culicidae |
| Fly | Culicidae | sp.3 | Culicidae |
| Fly | Culicidae | sp.4 | Culicidae |
| Fly | Empididae | sp.1 | Empididae |
| Fly | Empididae | sp.2 | Empididae |
| Fly | *Lasiopyrellia* | sp. | Muscidae |
| Fly | Muscidae | sp.1 | Muscidae |
| Fly | Muscidae | sp.2 | Muscidae |
| Fly | Muscidae | sp.3 | Muscidae |
| Fly | Muscidae | sp.4 | Muscidae |
| Fly | Muscidae | sp.5 | Muscidae |
| Fly | Muscidae | sp.6 | Muscidae |
| Fly | Muscidae | sp.7 | Muscidae |
| Fly | Muscidae | sp.8 | Muscidae |
| Fly | Muscidae | sp.9 | Muscidae |
| Fly | Muscidae | sp.10 | Muscidae |
| Fly | Muscidae | sp.11 | Muscidae |
| Fly | Muscidae | sp.12 | Muscidae |
| Fly | Muscidae | sp.13 | Muscidae |
| Fly | Muscidae | sp.14 | Muscidae |
| Fly | Muscidae | sp.15 | Muscidae |
| Fly | Muscidae | sp.16 | Muscidae |
| Fly | Muscidae | sp.17 | Muscidae |
| Fly | Muscidae | sp.18 | Muscidae |
| Fly | Mydidae | sp. | Mydidae |
| Fly | *Pliomelaena* | sp. | Tephritidae |
| Fly | *Pyrellia* | sp.1 | Muscidae |
| Fly | *Pyrellia* | sp.2 | Muscidae |
| Fly | Sarcophagidae | sp.1 | Sarcophagidae |
| Fly | Sarcophagidae | sp.2 | Sarcophagidae |
| Fly | Sarcophagidae | sp.3 | Sarcophagidae |
| Fly | *Stomoxys* | sp.1 | Muscidae |
| Fly | *Stomoxys* | sp.2 | Muscidae |
| Fly | Syrphidae | sp.1 | Syrphidae |
| Fly | Syrphidae | sp.2 | Syrphidae |
| Fly | Syrphidae | sp.3 | Syrphidae |
| Fly | Syrphidae | sp.4 | Syrphidae |
| Fly | Syrphidae | sp.5 | Syrphidae |
| Fly | Syrphidae | sp.6 | Syrphidae |
| Fly | Syrphidae | sp.7 | Syrphidae |
| Fly | Syrphidae | sp.8 | Syrphidae |
| Fly | Tabanidae | sp.1 | Tabanidae |
| Fly | Tabanidae | sp.2 | Tabanidae |
| Fly | Tabanidae | sp.3 | Tabanidae |
| Fly | Tabanidae | sp.4 | Tabanidae |
| Fly | Tabanidae | sp.5 | Tabanidae |
| Fly | Tabanidae | sp.6 | Tabanidae |
| Fly | Tabanidae | sp.7 | Tabanidae |
| Fly | Tabanidae | sp.8 | Tabanidae |
| Fly | Tabanidae | sp.9 | Tabanidae |
| Fly | Tabanidae | sp.10 | Tabanidae |
| Fly | Tabanidae | sp.11 | Tabanidae |
| Fly | Tachinidae | sp.1 | Tachinidae |
| Fly | Tachinidae | sp.2 | Tachinidae |
| Fly | Tachinidae | sp.3 | Tachinidae |
| Fly | Tachinidae | sp.4 | Tachinidae |
| Fly | Tephritidae | sp.1 | Tephritidae |
| Fly | Tephritidae | sp.2 | Tephritidae |
| Fly | Tephritidae | sp.3 | Tephritidae |

Supplementary Table S2: Species richness estimate and measures of sampling completeness

|  | Bee | Beetle | Fly | Wasp |
| --- | --- | --- | --- | --- |
| Observed species richness | 52 | 72 | 82 | 47 |
| Jack1 | 72.96 | 88 | 108.94 | 78.69 |
| Chao1 | 88.75 | 93.33 | 118.45 | 120.14 |
| ICE | 66.82 | 86.72 | 107.81 | 129.16 |

ICE(Incidence based coverage)
