# Supplementary material for: Asynchrony among insect pollinator groups and flowering plants with elevation
Source: Sci Rep. 2020 Aug 6;10:13268. doi: 10.1038/s41598-020-70055-5 (PMC7411018; doi:10.1038/s41598-020-70055-5)
Supplement: Supplementary file 2 — Supplementary Figure S1. [file 41598_2020_70055_MOESM2_ESM.docx]

**Asynchrony among insect pollinator groups and flowering plants with elevation**

***Opeyemi Adedoja^1,2^, Temitope Kehinde^3^, Michael J. Samways^1^**

^1^ Department of Conservation Ecology and Entomology, Stellenbosch University, South Africa

^2^Department of Conservation and Marine Sciences, Cape Peninsula University of Technology, South Africa

^3^Department of Zoology, Obafemi Awolowo University, Ile-Ife, Nigeria


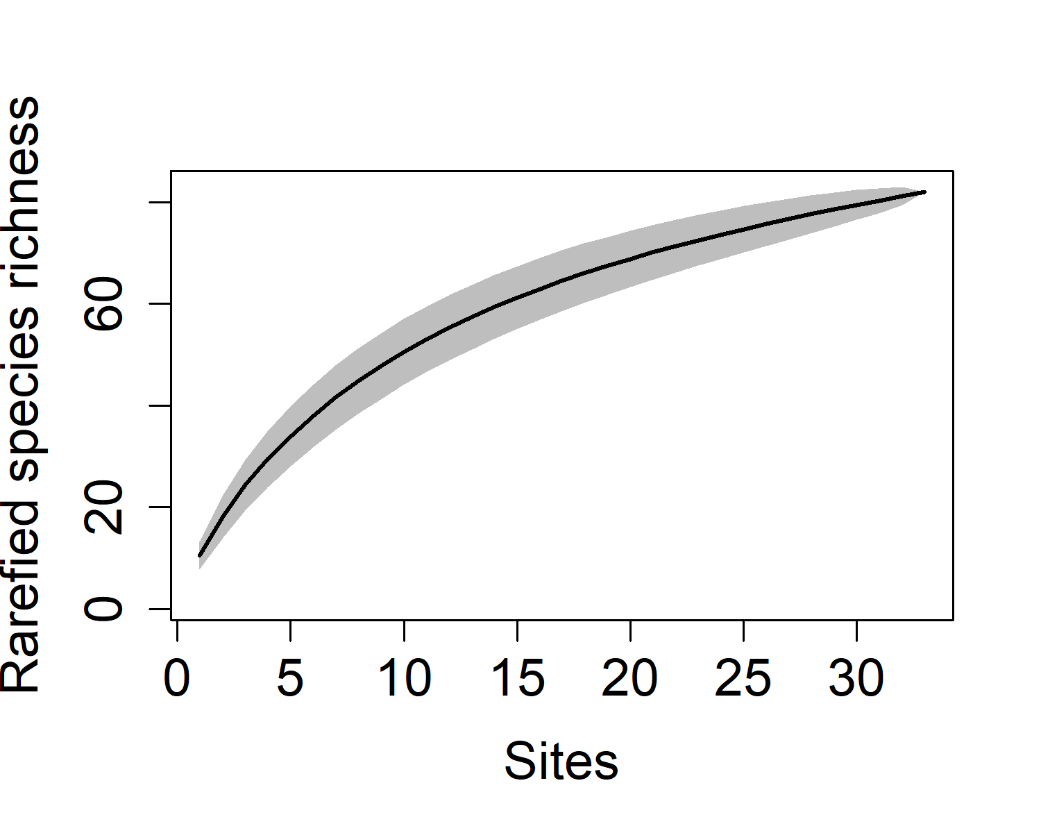


(c)


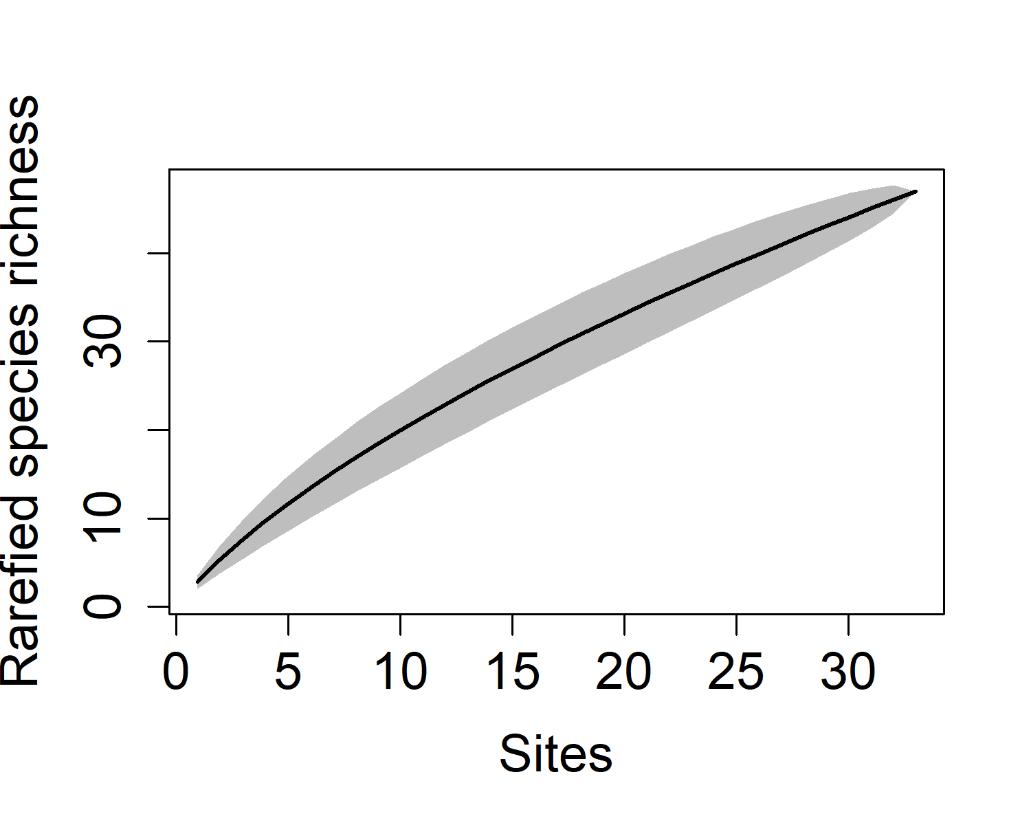


(d)


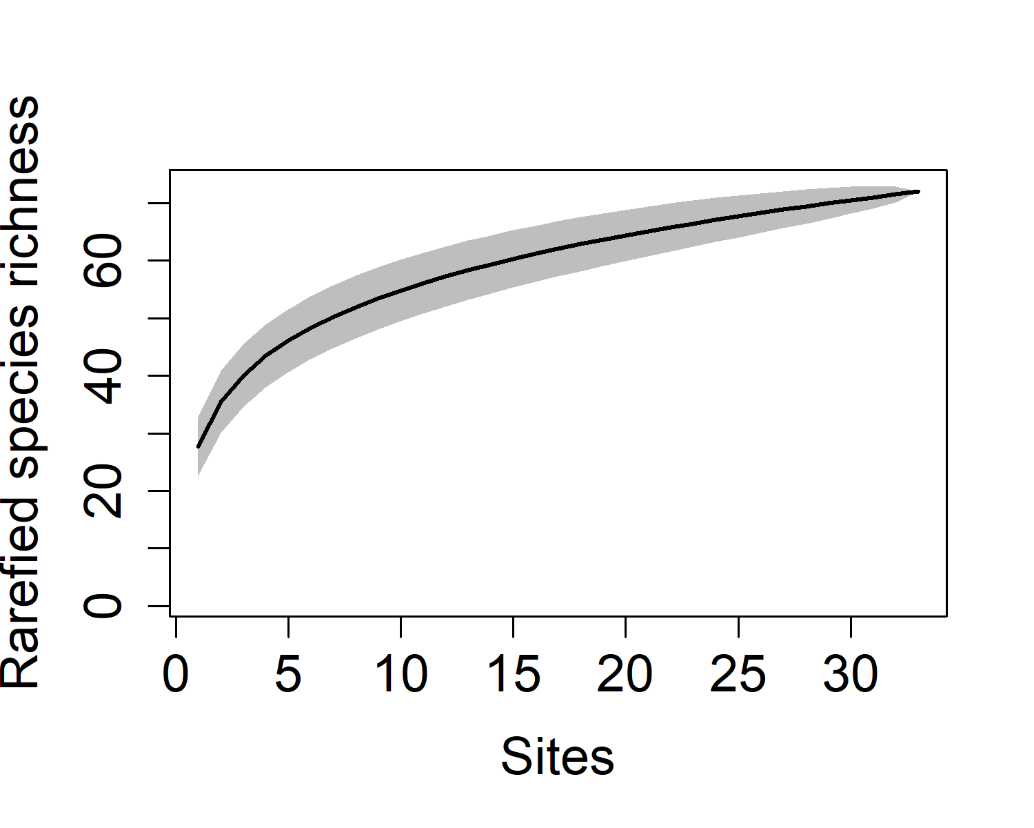


(b)


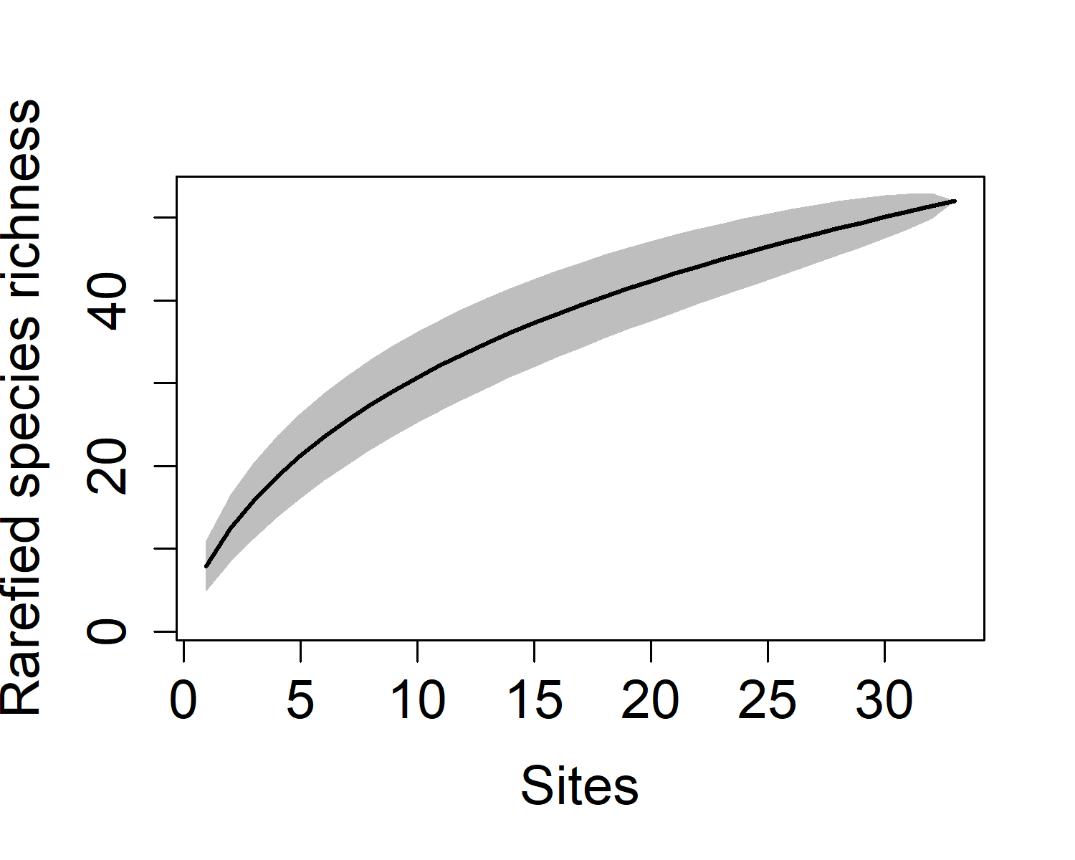


(a)

Supplementary Figure S1. Species richness rarefied curve for (a) bees (b) beetles (c) flies and (d) wasps sampled across sampling sites. The grey area represents the confidence interval from the standard error of estimates.
