# Supplementary material for: Asynchrony among insect pollinator groups and flowering plants with elevation
Source: Sci Rep. 2020 Aug 6;10:13268. doi: 10.1038/s41598-020-70055-5 (PMC7411018; doi:10.1038/s41598-020-70055-5)
Supplement: Supplementary file 3 — Supplementary Figure S2. [file 41598_2020_70055_MOESM3_ESM.docx]

**Asynchrony among insect pollinator groups and flowering plants with elevation**

***Opeyemi Adedoja^1,2^, Temitope Kehinde^3^, Michael J. Samways^1^**

^1^ Department of Conservation Ecology and Entomology, Stellenbosch University, South Africa

^2^Department of Conservation and Marine Sciences, Cape Peninsula University of Technology, South Africa

^3^Department of Zoology, Obafemi Awolowo University, Ile-Ife, Nigeria

**
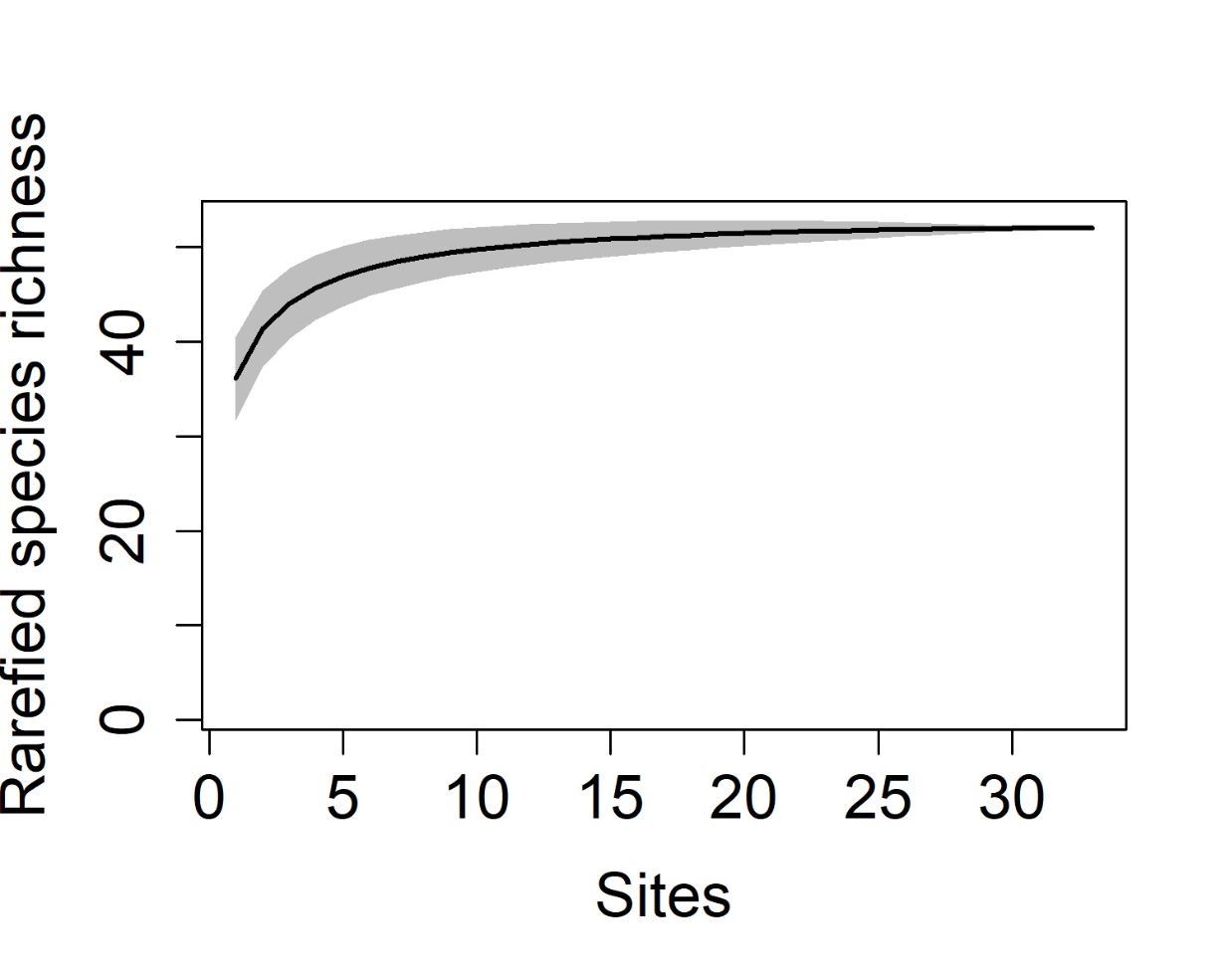
**

Supplementary Figure S2. Species richness rarefied curve for flowering plants sampled across study sites. The grey area represents the confidence interval from the standard error of estimates.
